# The relationship between human dignity and medication adherence in patients with heart failure

**Published:** 2017-06-18

**Authors:** Seyedeh Somayeh Amininasab, Hamideh Azimi Lolaty, Mahmood Moosazadeh, Vida Shafipour

**Affiliations:** 1 MSc Student of Intensive Care Nursing, Student Research Committee, School of Nursing and Midwifery, Mazandaran University of Medical Sciences, Sari, Iran.; 2 Assistant Professor, Department of Psychiatric Nursing, Psychiatry and Behavioral Sciences Research Center, Addiction Institute, Mazandaran University of Medical Sciences, Sari, Iran.; 3 Assistant Professor, Health Sciences Research Center, Faculty of Health, Mazandaran University of Medical Sciences, Sari, Iran.; 4 Assistant Professor, Department of Medical-Surgical Nursing, Nasibeh Nursing & Midwifery Faculty, Mazandaran University of Medical Sciences, Sari, Iran.

**Keywords:** *Human dignity*, *Medication adherence*, *Heart failure*, *Nursing*

## Abstract

Medication adherence is a behavior that is influenced by several factors, and maintaining patients’ dignity is an important issue that needs to be considered in the course of treatment**. **The present study aimed to determine the relationship between human dignity and medication adherence in patients with heart failure. This was a cross-sectional study. A total number of 300 patients with heart failure admitted to the Mazandaran Heart Center, Iran, participated in this study by census. Samples were selected based on inclusion criteria such as an HF diagnosis by a cardiologist for a minimum of 6 months, and taking at least one cardiac medication. Data were collected through demographic, clinical, human dignity, and medication adherence questionnaires over a period of three months in 2016. This study was approved by the Ethics Committee of Mazandaran University of Medical Sciences. Consents were obtained from patients and the medical center, and necessary explanations were given about the confidentiality of information prior to completing the questionnaires. The mean score of medication adherence was 5.82 suggesting low medication adherence among the patients, and the mean score of human dignity was 81.39. There was a negative relationship between medication adherence and threat to human dignity (r = - 0.6, *P* < 0.001), i.e., the higher the scores of threat, the lower the medication adherence of the patients. After adjusting the effects of potential confounding variables, there still was a correlation between medication adherence and the variables of human dignity and its dimensions. Based on the findings, an increase in patients’ dignity can enhance medication adherence, which can theoretically improve patients’ health and reduce frequent hospitalization.

## Introduction

Heart failure (HF) is a disorder of the heart structure and function that leads to failure in the transport of proper amounts of oxygen to tissues according to their metabolic needs. Clinically, HF is a syndrome in which patients demonstrate symptoms such as dyspnea, swollen ankles and fatigue, and signs such as jugular venous pressure, and crackles in the lungs as a result of abnormal heart structure and function (1). HF is a growing heart problem in the United States (2) where it is estimated to be affecting more than 5.8 million people; moreover, there are 23 million people suffering from the disorder worldwide. HF prevalence is expected to increase to 46% by 2030, i.e., about 8.5 million people (3). Based on the studies in Iran, 25% of the patients admitted to cardiology wards are diagnosed with HF, which indicates that it is a pandemic disease (4). Readmission of patients with HF dramatically increases patients’ costs, and one of the main reasons for this is their lack of adherence to the prescribed medication regimen(5).

Adherence to medication regimen is a disease-related behavior that predicts successful treatment outcomes and reduces the adverse effects and severity of the disease (6). Medication adherence may be defined as compliance with all medication orders (7, 8) or consumption of more than 80% of the prescribed medicines, but the definite cause is not certain (9). Medication non-adherence is the failure to comply with healthcare recommendations and refusal to follow the medication regimen by individuals (6). It is a complex behavioral process influenced by many factors, and according to the WHO model, its predictors include 5 dimensions: 1) Healthcare system factors (health team members’ communication skills and patient satisfaction); 2) Patient-related factors (age, gender, beliefs and attitudes); 3) Socioeconomic factors (education, income and social support); 4) Treatment-related factors (complexity and side effects of the treatment); and 5) Condition-related factors (illness severity and comorbidities) (10). Identification of these factors improves patients’ adherence to treatment regimens, and enhances care providers decisions and performances (3). Despite its importance, patients’ adherence to medication regimen is 25% - 50%, which can lead to adverse consequences, including poor clinical results, readmission, and increased healthcare costs (11). It is estimated that adherence to the prescribed regimen could prevent 54% of HF cases (5). According to WHO, the mean medication adherence of patients with chronic diseases is 50% in developing countries. Medication non-adherence remains a major barrier to increasing the effectiveness of treatments (12) and results in poor treatment, imposes billions of dollars of excess costs a year, causes 125,000 deaths per year, and is the reason for 10% of all hospitalizations in the United States (13).

Human dignity is considered one of the most important features and ethical concerns in healthcare and nursing care. There are two types of human dignity: absolute and relative dignity. The former pertains to the essence of each creature, and the latter is influenced by the society and human relations (14). Dignity is described as a human characteristic in professional care (15). Based on the definition of dignity, all human beings are created free and equal in rights (4). In fact, respect for human dignity is observance of individuals’ basic rights in different environments (16). Human dignity is related to the property of being a human, and is of the essence in healthcare systems (17). It has a prominent position in the studies and discussions on healthcare, as well as health equity (18). Patients may be among the most vulnerable social groups, as they not only have lost their physical abilities, but also are under the particular psychological, social and economic pressures imposed by their illness. Compared to other chronic diseases, heart failure has a more noticeable impact on the individual’s performance in social, family, and marital relationships (19). The primary goal of HF care is to increase patients’ life expectancy (20). Care providers need to maintain an honest communication with the patients and respect their personal rights and professional values ​​such as human dignity, and be sensitive to the existing differences (21). Respect for patients’ dignity plays an important role in their treatment and increases their quality of life (9, 18). Moreover, healthcare system related factors such as communication between patient and provider could enhance medication adherence (22), and improve patient dignity (23). As a result, the researchers investigated the probable relationship between preservation of human dignity and incentives to increase patient adherence to medical recommendations. Furthermore, a literature review revealed studies about the relationship between medication adherence and cognitive impairment in the elderly (11), self-management interventions (24), awareness of disease (25), health literacy (26), anxiety and psychosomatic disorders (13), and patients’ beliefs and attitudes (27); however, the researchers were unable to find studies on the relationship between human dignity and medication adherence. Therefore, the present study focused on the importance of maintaining patients’ dignity and the essential role of medication adherence in the successful treatment of HF. Thus, the researchers aimed to determine the relationship between human dignity and medication adherence in patients with heart failure.

## Methods

This cross-sectional study recruited patients with heart failure on their first day of admission to CCU wards of Mazandaran Heart Center in 2016. Census sampling method was used to select all patients with heart failure that met the inclusion criteria. In order to determine the sample size, the number of patients admitted with heart failure in a month per year was first established through hospital medical records. Sampling was carried out based on that figure for three months. Then, the sample size (α = 0.01, β = 0.1, r = 0.25) was determined at 300 patients using GPOWER software. Inclusion criteria were: being older than 18 years, Iranian nationality, residing in Sari, Mazandaran province, speaking Persian, HF diagnosis from a cardiologist for a minimum of 6 months, taking at least one cardiac medication, classes II and III of the New York Heart Association classification, ejection fraction higher than 30%, lack of sensory perception disorder or mental retardation, full consciousness and the ability to answer questions.

Data were collected by demographic and clinical questionnaires, Patient Dignity Inventory, and the Morisky Medication Adherence Scale (MMAS-8) revised in 2008. Clinical and demographic questions included age, sex, marital status, income, number of children, education, occupation, location of residence, comorbidities including diabetes and hypertension, and the number of pills consumed per day. In order to measure human dignity, the Patient Dignity Inventory (PDI) developed by Chochinov et al. in 2008 was used. The reliability and validity of the PDI were reported as Cronbach’s alpha coefficient of 93% and 0.85 (28). Abbas-Zadeh et al. in 2015 translated the PDI into Persian and evaluated its validity and reliability in patients with coronary heart diseases such as myocardial infarction, heart failure, and acute coronary syndrome (Cronbach’s alpha = 0.85) (29). The questionnaire consists of 25 items in five domains, including distress symptoms (items: 3, 5, 6, 7, 8, 9), existential distress (items: 4, 11, 12, 13, 14, 18, 19), dependency (items: 1, 2, 10, 20), peace of mind (items: 15, 16, 17) and social support (items: 21, 22, 23, 24, 25). Questions were scored in Likert scale from 1 to 5 (1: Not a problem; 2: A slight problem; 3: A problem; 4: A major problem; and 5: Overwhelming problem). The scores ranged from 25 to 125, with 25 showing the highest, and 125 showing the lowest degree of dignity, and the score of 75 and above represented a threat to the patients’ dignity. The score of 18 in distress symptoms (score range: 6 - 30), 9 in peace of mind (score range: 3 - 15), 12 in dependency (score range: 4 - 20), 15 in social support (score range: 5 - 25), and 21 in existential distress (score range: 7 - 35) represented a threat to the patients’ dignity. Reliability was determined at 0.87 through the interclass correlation coefficient of Cronbach’s alpha coefficient.

Medication adherence was calculated by Morisky Medication Adherence Scale (30). The 8-items form was validated by Rashedi et al. in 2011, and the Cronbach’s alpha was reported to be 0.83 (31). This self-report scale consists of 7 items answered with yes or no, and 1 item with a 5-point Likert scale (never = 0, rarely = 1, sometimes = 2, often = 3, always = 4). The minimum score was 0 and the maximum score was 11. The cut-off point was 6 and a score less than 6 was considered medication non-adherence (31). 

As the first step of the research, the participants were briefed on the purpose of the study, received instructions on how to complete the questionnaires, and were assured of the confidentiality of their responses. Subsequently, the questionnaires were completed after obtaining patients’ consent. Data were analyzed in SPSS version 16 using descriptive statistics and Pearson’s correlation test.

## Results

Based on the results obtained in this study, the participants’ mean age was 64.15, men and women equally comprised the study population, most (50.3%) were illiterate, and 49.3% had poor income (Table 1). 

**Table 1:** Personal characteristics of the patients with heart failure

Personal Characteristics		Frequency	Percent
Age Group	35 - 44	14	4.7
	44 - 54	50	16.7
	55 - 64	99	33
	56 - 74	72	24
	> 74	65	21.7
Gender	Male	150	50
	Female	150	50
Education	Illiterate	151	50.3
	Under high school diploma	88	29.3
	Diploma and advanced diploma	47	15.7
	Bachelor’s degree and above	14	4.7
Marital Status	Single	1	0.3
	Married	293	97.7
	Divorced	3	1
	Widowed	3	1
Occupation	Employee	34	11.3
	Laborer	30	10
	Farmer	25	8.3
	Housewife	135	45
	Self-employed	31	10.3
	Retired	45	15
Number of Children	Less than 2	30	10
	2 - 5	187	62.3
	More than 5	83	27.7
Place of Residence	Urban	191	63.7
	Rural	109	36.3
Income	Poor	148	49.3
	Fair	136	45.3
	Good	16	5.3
Frequency of Hospitalization	Less than 2	46	15.3
	2 - 5	161	537
	More than 5	93	31
Comorbidities	Diabetes	69	23
	Hypertension	93	31
	Diabetes and hypertension	77	25.7
	No underlying disease	61	20.3
Severity of the Disease (LVEF)	50 - 60	124	41.3
	40 - 50	110	36.7
	30 - 40	66	23
The Number of Pills Consumed per Day	Less than 5	86	28.7
	5 - 10	143	47.7
	More than 10	71	23.7

LVEF=left ventricular ejection fraction

The mean medication adherence in this study was 5.82 in patients with HF, which is considered low according to the research tool. The mean score of human dignity was 81.39. Table 2 shows the mean, standard deviation, and range of the PDI scores for human dignity and its dimensions, that is, distress symptoms, peace of mind, dependency, social support and existential distress. According to PDI, a higher score represents a greater threat to patients’ dignity. There was a negative relationship between medication adherence and threat to human dignity (correlation coefficient r = - 0.6, significance level *P* < 0.001), (Table 2). 

**Table 2:** Mean and standard deviation and correlation between medication adherence and human dignity, and the factors threatening it

Variable	Questionnaire Range	Obtained Range	Mean	SD	Correlation Coefficient (r)	*P*-Value
Human Dignity and its Dimensions	25 - 125	37 - 125	81.39	16.52	- 0.66	< 0.001
Distress Symptoms	6 - 30	8 - 30	19.06	4.21	- 0.65	< 0.001
Peace of Mind	3 - 15	4 - 15	9.38	2.32	- 0.61	< 0.001
Dependency	4 - 20	6 - 20	14.37	2.38	- 0.66	< 0.001
Social Support	5 - 25	5 - 25	14.95	3.45	- 0.62	< 0.001
Existential Distress	7 - 35	11 - 35	23.62	5.03	- 0.60	< 0.001

In other words, the higher the score of threat to dignity, the lower the medication adherence. Even after adjusting the potential confounding variables in this study (age, sex, marital status, place of residence, occupation, education, number of children, frequency of hospitalization, comorbidities, ejection fraction severity, and the number of pills consumed per day), there still was a correlation between the variables of human dignity and its dimensions, and medication adherence (Table 3).

**Table 3:** Partial correlation between medication adherence and human dignity (after adjustment of confounding variables)

Variable	Correlation Coefficient (r)	*P*-Value
Human Dignity and its Dimensions	- 0.66	< 0.001
Distress Symptoms	- 0.65	< 0.001
Peace of Mind	- 0.49	< 0.001
Dependency	- 0.65	< 0.001
Social Support	- 0.60	< 0.001
Existential Distress	- 0.59	< 0.001

## Discussion

The main finding in this study was the significant relationship between medication adherence and human dignity and its dimensions (distress symptoms, peace of mind, dependency, social support and existential distress), so that even by eliminating potential confounding variables, the relationship still existed. The patients in this study had reduced human dignity, and consequently low levels of medication adherence were reported.

Even though studies have been conducted on the dimensions of human dignity and medication adherence separately, none were found on the connection between the two on available databases. This research revealed a significant relationship between medication adherence and distress symptoms, which is in line with the findings of previous studies on the relationship between psychological distress and medication adherence. The following authors have worked in this regard: Alosco et al. studied the relationship between cognitive dysfunction and treatment adherence in patients with heart failure (32). Gehi et al. and van der Wal et al. investigated the relationship between depression and medication adherence (33, 34); and Schweitzer et al. examined the impact of psychological factors on treatment adherence behavior in patients with heart failure (35). In addition, some studies were conducted on the relationship between distress symptoms and medication adherence in patients with chronic diseases (36), acquired immune deficiency syndrome (AIDS) (37, 38), epilepsy (39), and children receiving transplants (40). A few studies showed that there were no significant associations between depression (41) and anxiety (13, 42), and medication adherence, but overall it seems that the higher the distress is, the lower the medication adherence will be.

In this study, there was a significant relationship between the peace of mind dimension of patient dignity and medication adherence in HF patients. This is consistent with the findings of another study, which showed that respect for the patients and their experiences and interests increased their confidence and consequently medication adherence (43). 

There was a significant relationship between the dependency dimension and medication adherence in this study. Similarly, Maeda et al. (20) and Criswell et al. (44) revealed a relationship between self-efficacy and medication adherence among heart failure, and hypertension patients, respectively. Heydari et al. examined “self-concept” in the two domains of challenges and threats. They reported that threat to self-concept invoked a response based on a feeling and led to an individual’s non-adherence to treatment regimens (5). 

The present study showed that there was a significant relationship between social support and medication adherence, which is backed by studies on different patients (20, 44-50). On the other hand, there are studies on patients with heart diseases that found no significant relationship between social support and medication adherence (12, 51-53). Beals et al. even reported that social support decreased medication adherence (54). These differences could point to certain psychological aspects of social support that cause some patients not to adhere to medications in order to gain support and fulfill their psychological needs.

The findings of this study demonstrated a significant relationship between the existential distress dimension of patient dignity and medication adherence. This dimension is associated with the reduced ability of patients to do their daily activities as a major threat to human dignity. A study showed that diminished performance and activity had a significant effect on medication adherence in patients with HF (32). Similarly, other studies on patients with diabetes and hyperlipidemia indicated that reduced strength and performance was related to low medication adherence (32, 55). Based on these results, the more serious the patients’ disabilities are, the lower their medication adherence will become.

This study was conducted in only one medical center, and it is recommended to perform further studies in multiple centers in order to investigate the generalizability of the findings. 

## Conclusion

Medication adherence is a multidimensional behavior influenced by several factors. Therefore, care providers need to first identify these factors and then consider them in training and treatment planning for patients in order to increase their medication adherence. Based on the results, it is recommended to maintain patients’ dignity as an important factor that should be considered in the course of treatment, and can improve patients’ recovery and their return to normal life***. ***

It is therefore recommended to study the effects of dignity therapy on patients with heart failure, and the role of education in improving medication adherence further. Also, it is suggested that similar studies be conducted on patients suffering from other chronic diseases such as diabetes or multiple sclerosis, and those who have undergone surgery or are receiving hemodialysis. 
